# Active self-treatment of a facial wound with a biologically active plant by a male Sumatran orangutan

**DOI:** 10.1038/s41598-024-58988-7

**Published:** 2024-05-02

**Authors:** Isabelle B. Laumer, Arif Rahman, Tri Rahmaeti, Ulil Azhari, Hermansyah, Sri Suci Utami Atmoko, Caroline Schuppli

**Affiliations:** 1 Development and Evolution of Cognition Research Group, Max Planck Institute of Animal Behavior, Konstanz, Germany; 2 Department of Biology, Graduate Program, Faculty of Biology and Agriculture, Universitas Nasional, Jakarta, 12520 Indonesia; 3 SUAQ Project, Medan, Indonesia; 4 Yayasan Ekosistem Lestari (YEL), Medan, Indonesia; 5 Fakultas Biologi, Universitas Nasional, Jakarta, Indonesia

**Keywords:** Animal self-medication, Therapeutic topical application, Phytotherapy, Great apes, Ethnomedicine, Ecology, Evolution, Zoology

## Abstract

Although self-medication in non-human animals is often difficult to document systematically due to the difficulty of predicting its occurrence, there is widespread evidence of such behaviors as whole leaf swallowing, bitter pith chewing, and fur rubbing in African great apes, orangutans, white handed gibbons, and several other species of monkeys in Africa, Central and South America and Madagascar. To the best of our knowledge, there is only one report of active wound treatment in non-human animals, namely in chimpanzees. We observed a male Sumatran orangutan (*Pongo abelii*) who sustained a facial wound. Three days after the injury he selectively ripped off leaves of a liana with the common name Akar Kuning (*Fibraurea tinctoria*), chewed on them, and then repeatedly applied the resulting juice onto the facial wound. As a last step, he fully covered the wound with the chewed leaves. Found in tropical forests of Southeast Asia, this and related liana species are known for their analgesic, antipyretic, and diuretic effects and are used in traditional medicine to treat various diseases, such as dysentery, diabetes, and malaria. Previous analyses of plant chemical compounds show the presence of furanoditerpenoids and protoberberine alkaloids, which are known to have antibacterial, anti-inflammatory, anti-fungal, antioxidant, and other biological activities of relevance to wound healing. This possibly innovative behavior presents the first systematically documented case of active wound treatment with a plant species know to contain biologically active substances by a wild animal and provides new insights into the origins of human wound care.

## Introduction

In the early 1960s Jane Goodall first described the presence of whole leaves in the feces of chimpanzees (*Pan troglodytes*) at Gombe Stream, Tanzania^1^. By the late 1990s, this behavior, now called whole leaf swallowing, was documented at several African great ape study sites, along with bitter pith chewing, and demonstrated to have therapeutic, anti-parasitic functions^2^. Since then, various forms of self-medication have been observed in wild great apes (e.g.,^2–6^). Some of the most detailed evidence for animal self-medication comes from research in primates (e.g.,^1–12^).

Animal self-medication is now divided into five categories^2,4^: (1) sick behaviors, such as anorexia; (2) avoidance behaviors, such as avoiding e.g. feces, contaminated food or water; (3) prophylactic behaviors, such as routine consumption of foods with preventive or health maintenance effects; (4) therapeutic behaviors, defined by the ingestion of a small amount of a biologically active or toxic substance with no or little nutritional value for the curative treatment of a disease or its symptoms, and; (5) therapeutic topical application of pharmacologically active plants onto the body for the treatment of external health conditions or placement of such species in the nest as a fumigant or insect repellent^13^. Several of these behaviors can be found in wild apes^2^.

While sick and avoidance behavior (category 1 and 2) can be regularly observed in non-human animals (e.g.^14^), self-medication in the form of ingestion of specific plant parts (prophylactic and therapeutic behavior, category 3 and 4) is widespread, albeit exhibited at low frequencies (e.g.,^15^, but see^16^). So far, leaf swallowing has been reported in chimpanzees (*Pan sp.*; e.g.,^7,9,16,17^), bonobos (*Pan paniscus*^3^), gorillas (e.g. *Gorilla beringei graueri*^18^), and in only one Asian ape species, the white-handed gibbon (*Hylobates lar*)^19^. Another study reported the consumption of plant species directly related to the occurrence of parasite infections in individual orangutans (*Pongo sp.*), but not correlated with the plant’s distribution in the environment^20^. Another therapeutic self-medicative behavior seen in chimpanzees is bitter pith chewing of *Vernonia amygdalina* to treat worm infection^8,10,13^. Despite the plant’s year-round availability, the behavior is highly seasonal, peaking during the rainy season when worm infections also peak^8,13^. Interestingly, as *Vernonia amygdalina* is not evenly distributed in their home range, the apes often need to actively adapt their usual travel routes to gain access to the plant^13^.

Among Bornean orangutans (*Pongo pygmaeus*) there are several reports proposing the intentional ingestion of specific plant species also used in ethnomedicine for their medicinally active properties. In Sabah, Malaysia, a 4- to 5-year-old severely wounded female Bornean orangutan was observed eating ginger leaves and stem (Zingiberaceae)^21^. Ginger is known as a traditional medical plant against inflammation with antibacterial, antiviral, antifungal properties^22–25^. In 7 years of observation, no other individual, except two flanged males was ever observed feeding on the same ginger species at that study site. The researchers concluded that the juvenile may have attempted to treat itself with these plants. Another study, which interviewed 13 traditional healers from Central Kalimantan, showed that Bornean orangutans feed on the same plant parts from two plant species (*Uncaria gambir Roxb* and *Pternandra galeata Ridl*), used by traditional healers for treating internal illness, tumors, and haemorrhage^26^. Additionally, they observed a female Bornean orangutan selectively choosing young leaves of *Mezzetia sp.*, the pulp of *Dyera lowii* and *Ilex cymosa*, and leaves of Belang Handipek (*Scolopia macrophylla)*^27^. This plant combination is used in ethnomedicine as a prevention against fatigue^27^. Despite these reports, overall, evidence of plant consumption for self-medication in orangutans is still limited.

Reports of the topical application of plants or insects to one’s own body (category 5) are found in a limited number of taxa, but the evidence for medicinal benefits remain mostly anecdotal (e.g.^28–38^). However, there is growing evidence for the application of biologically active plant compounds to the skin in orangutans. At Sabangau peat swamp forest in Central Kalimantan, two adult female and one adolescent female Bornean orangutans were observed chewing leaves of *Dracaena cantleyi* for three to five minutes and then rubbing the resulting green-white lather onto their arms and legs for up to 35 min^11^. Ten years later, a follow-up study confirmed the same behavior in six additional adult females and one flanged male of the same population (the lather was similarly applied and massaged into the skin for up to 45 min^6^). The behavior appeared to be intentional as only specific body parts were treated, the behavior was repeated several times until the hair was fully wet and the entire process took a considerable amount of time^6,11^. Orangutans were never observed ingesting the leaves^6^. *Dracaena cantleyi* is a medicinal plant used by indigenous people for several medical treatments including sore muscles, joint or bone pain^6^, pain after a stroke^6^ and swelling^11^. Indeed, pharmacological analyses revealed that *Dracaena cantleyi* inhibits TNFα-induced inflammatory cytokine production thereby acting as an anti-inflammatory agent^6^.

There are some brief anecdotal mentions of chimpanzees using leaves (plant species unknown) to wipe blood from their wounds^39,40^. Active wound treatment with a substance has only recently been documented for the first time in a great ape species. Chimpanzees of the Rekambo community (*Pan troglodytes troglodytes*) in the Loango National Park, Gabon, were observed applying insects to their own wounds (n = 19) and to the wounds of conspecifics (n = 3)^5^. The five adult males, one adult female, and one juvenile female applied the insects in the same sequence: they caught a dark-colored, winged insect approximately 5 mm in size (unidentified at the time of publication), immobilized it by squeezing it between the lips, then applied the insect to the wound moving it with their mouth or finger, then removed it. The last two steps were usually repeated several times. Further research is needed to investigate the efficiency of this behavior. Active wound treatment has also been described in a captive capuchin monkey, that was observed grooming her vaginal area and four of her own wounds with a sugar-coated tool^41^. However, as the authors noted that the capuchin was used to having her wounds treated with an antibacterial salve topically applied by caregivers.

We here report for the first time active wound treatment with a known biologically active plant substance by a male Sumatran orangutan in the wild, and discuss the hypothesis that this may be a form of self-medication to treat a wound and possibly prevent infection and accelerate wound healing.

## Methods

### Study site

The observations took place in the Suaq Balimbing research area (N 3° 02.873′, E97° 25.013′), a part of the Gunung Leuser National Park in South Aceh, Indonesia. The research area is approximately 350 ha and consists mainly of peat swamp forest. Since 1994, the wild Sumatran orangutans (*Pongo abelii*) at Suaq have been the subjects of non-invasive, almost exclusively observational research.

### Subject information

A male Sumatran orangutan named Rakus was first observed in March 2009. At that time, Rakus was an unflanged male (i.e., adult but without secondary sexual characteristics^42^) and was estimated to be born in the late 1980s. He is either a resident to the area or a frequent visitor^43–45^. Rakus went through a secondary growth spurt in 2021 and has been a fully flanged male since August 2021.

### Data collection procedure

Data on orangutans at Suaq Balimbing are collected using all-day focal follows. Data collection starts when an individual is found or when they leave their night nest in the morning until they build a new night nest in the evening. Data is collected at two-minute intervals following standardized protocols for orangutan behavioural observations. Furthermore, all rare behaviors are described in detail on an all-occurrence basis in the notes section of the data sheets.

Rakus was a focal individual from June 22 (on the day this fresh wound was first noted) to June 26, from June 28 to 30, on July 5, July 19, and August 5, 2022. The wound treatment data was collected on June 25, 2022, and described in detail in the notes section of the data sheets. Unfortunately, no photos or videos were taken of the wound treatment.

We took detailed pictures of the plant specimen that was used by the orangutan to ensure reliable identification (see Fig. 2). However, due to a lack of the necessary permits, we were unable to collect and store a physical sample of the specimen. The pictures of the specimen were compared to the site’s detailed picture-based herbarium which was established at the beginning of the research activities at Suaq Balimbing via samples collected at the site in partnership with the National Herbarium of Indonesia and the National University of Indonesia (UNAS).

### Ethical guidelines

The data collection in wild orangutans was strictly observational and collected without any interaction with the study animals. The research protocols were approved by the Ministry of research and technology (RISTEK; research permit no. 152/SIP/FRP/SM/V/2012 and following) and complied with the legal requirements of Indonesia.

## Results

**Figure 1:**
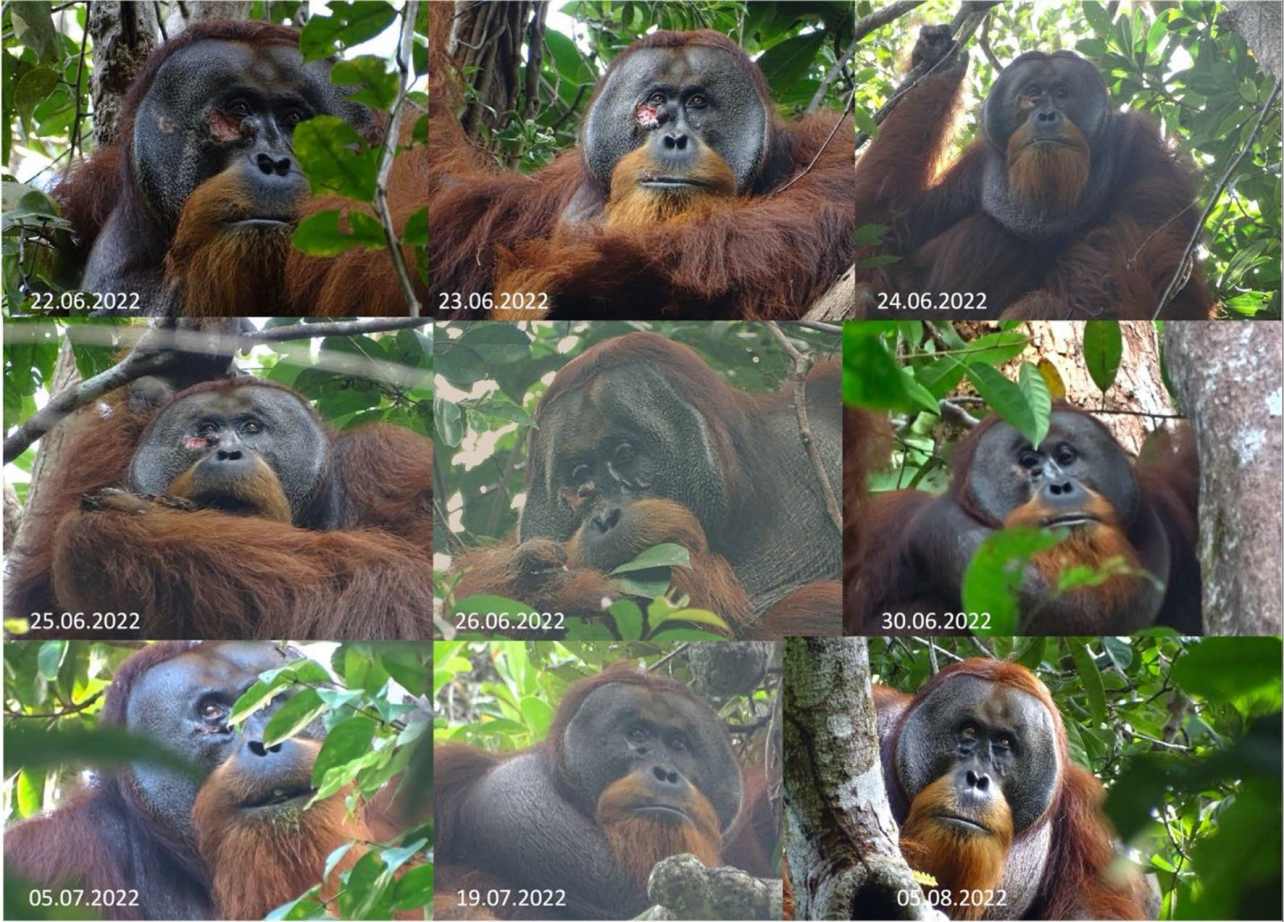
Process of wound healing. Rakus fed on and later applied the masticated leaves of *Fibraurea tinctoria* to his facial wound on June 25. On June 26 he was again observed feeding on *Fibraurea tinctoria* leaves (see photo). By June 30 the wound was closed and by August 25 was barely visible anymore.

On June 22, 2022, our research team (including UA) first noticed that Rakus had a fresh wound on his right flange (see Fig. 1, see movie S1) and inside his mouth, first visible when he emitted a long call; see movie S2). How he got the wound is unknown, however typically flanged males acquire these kinds of wounds during fights with other flanged males. Vocal evidence of a fight between orangutan males was reported earlier on the same day of this observation.

**Figure 2:**
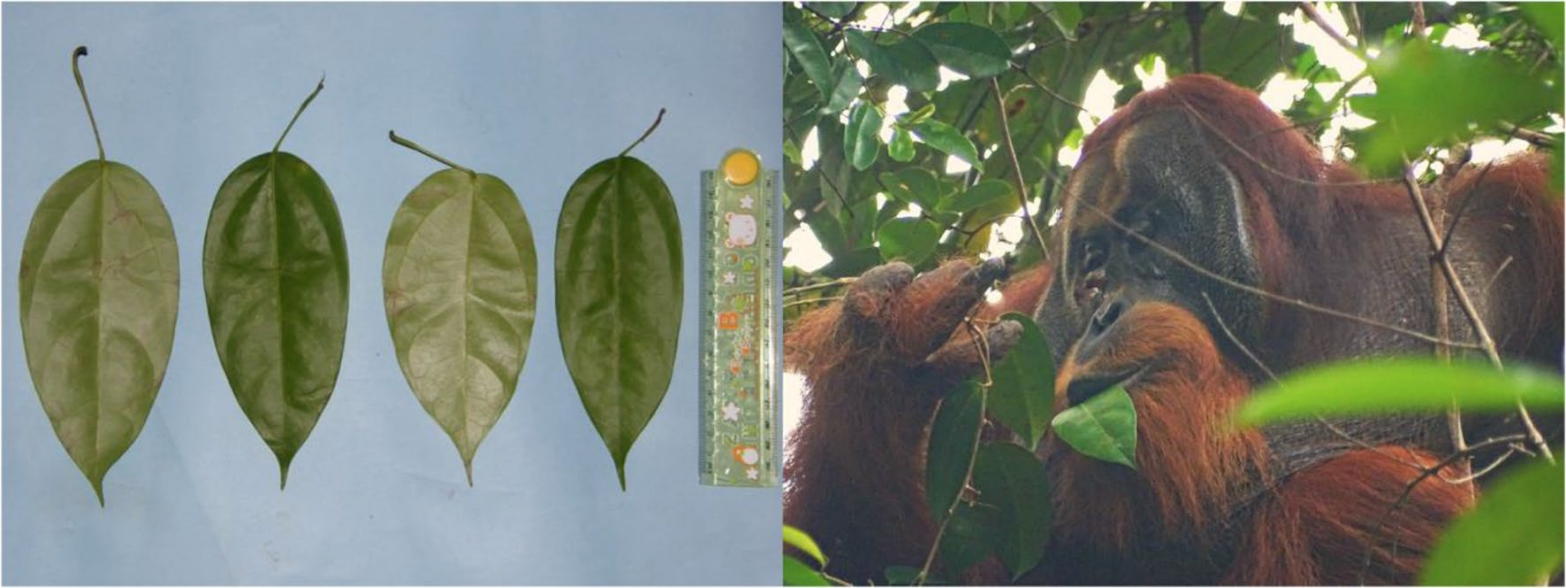
Left: Pictures of *Fibraurea tinctoria* leaves. The length of the leaves is between 15 to 17 cm. Right: Rakus feeding on *Fibraurea tinctoria* leaves (photo taken on June 26, the day after applying the plant mesh to the wound).

On June 25 at 11:16, Rakus started feeding on the stem and the leaves of the liana of *Fibraurea tinctoria* (see Fig. 2), also known as ‘Akar Kuning’ (for other synonyms and classification of the plant, please see SI, Table S1), which is part of the orangutans’ diet in this area. The liana is rarely eaten (0.3% of all feeding scans, n = 390′000), but 47 out of a total of the 132 orangutans on which we have collected feeding data were observed consuming its leaves, fruits, or parts of the stem. Thirteen minutes after Rakus had started feeding on the liana, he began chewing the leaves without swallowing them and using his fingers to apply the plant juice from his mouth directly onto his facial wound. This behavior was repeated several times and lasted seven minutes. After this period, at 11:36, flies of an unknown species appeared on the wound. Rakus then smeared the entire wound with the plant pulp until the red flesh was fully covered with the green leaf material. He then continued feeding on this plant for a total of 34 min. The next day (June 26), but not on any other of the following observation days (June 28, 29, 30; July 5, 19, 20), he ate leaves of *Fibraurea tinctoria* again for two minutes (pictures (Figs. 1 and 2) and one video (see movie S1) were taken on June 26th while he was feeding on *Fibraurea tinctoria*).

Observations over the following days did not show any signs of the wound becoming infected (see photos taken on June 26 and 28; Fig. 1) and by June 30 the facial wound was already closed (see Fig. 1 and movie S1). By July 19, 2022, the wound appeared to have fully healed and only a faint scar remained (see Fig. 1).

Furthermore, UA (who collected the focal data) noted that Rakus rested more than usual after being wounded, which may positively affect wound healing as growth hormone release, protein synthesis and cell division are increased during sleep^46,47^. When considering the percentage of time spent resting (based on 2-min interval activity scans) between January 30, 2021 (since then he has been considered a flanged male; this is important to consider, as flanged males in general rest more than unflanged males^12,48^) to February 22, 2023, we noticed that resting time increased after being wounded (June 22, 2022, to July 20, 2022; mean = 33 ± 17.2%) compared to the time before (January 30, 2021, to November 8, 2021; mean = 14.8 ± 7.4%) and that resting time decreased again after the wound had closed (August 5, 2022, to February 22, 2023; mean = 23.6 ± 12.9%; see SI, Figure S1). He spent more than 50% the day resting after he was found with the fresh wound (June 23, 2022; 52.2% of resting), the day after wound treatment (June 26, 2023; 51%), and four days after treatment (June 29, 2023; 54.3%; see SI, Fig. S1).

## Discussion

To the best of our knowledge, this study is the first systematic documentation of the putative active wound treatment with a biologically active plant substance in great apes and other non-human species. In this study, the flanged male orangutan Rakus was observed to selectively detach, chew, and repeatedly apply the chewed leave juice directly on his three-day-old facial wound for several minutes and covered the entire wound with a chewed-up leaf mash. Additionally, Rakus rested more when the wound was fresh compared to before and after wounding.

The treatment of human wounds was most likely first mentioned in a medical manuscript that dates back to 2200 BC, which included cleaning, making plasters and bandaging of wounds^49^. One of the earliest known wound care products used by the Sumerians, Greek, Mayans and Egyptians were oil, herbs, maggots, beer, vinegar, wine, green paint containing copper and honey^49,50^.

*Fibraurea tinctoria*, has other generic names such as Akar Kuning (Central Kalimantan), Akar Palo (Aceh), and Yellow Root (East Kalimantan^51^). It is an evergreen, climbing plant in the family Menispermaceae, with a broad distribution across Mainland China, Indonesia, Malaysia, Thailand, Vietnam and other areas of Southeast Asia^52,53^, and is known for its analgesic, antipyretic, antidote, and diuretic effects, and is used in traditional medicine to treat condition such as dysentery, diabetes, and malaria^51,54,55^. All plant parts have been reported to be used for these medical applications, including leaves, stems, roots and bark^54^.

Pharmacological analysis of the plant’s chemical compounds shows the presence of furanoditerpenoids^56^, a special group of diterpenoids composed of one or more aromatic furan rings (with four carbon atoms and one oxygen), which are reported to have antibacterial, anti-inflammatory, anti-fungal, antioxidant, and anticarcinogenic biological activities^55,56^. *Fibraurea tinctoria* also has a high concentration of protoberberine alkaloids, which have anti-inflammatory, analgesic, anticonvulsant, antiamnesic, narcotic, antiarrhythmic, antihemorrhagic, hypotensive, antioxidant, antitumoral, antidiuretic, antiulcer, and muscle relaxant properties^57^. It also contains jatrorrhizine (antidiabetic, antimicrobial, antiprotozoal, anticancer, and hypolipidemic properties; reviewed in^58^) and palmatine (anticancer, antioxidation, anti-inflammatory, antibacterial, antiviral properties; reviewed in^59,60^). Among 38 plants used in ethnomedicine and grown in South Vietnam, *Fibraurea tinctoria* showed the highest activity tested for antimalarial effects^61^. It has also been shown that the leaves and stems of *Fibraurea tinctoria* inhibit the growth of several bacteria species, including *Bacillus cereus*, *Staphylococcus aureus,* and *Escherichia coli*^53,62^. *Fibraurea tinctoria* also showed a significant anti-inflammatory effect in reducing mouse paw edema^55^.

Like all self-medication behavior in non-human animals, the case reported in this study raises questions about how intentional these behaviors are and how they emerge. Similar to plant ointment behavior in Bornean orangutans^6,11^, the behavior of the Sumatran flanged male orangutan reported here appeared to be intentional as (I) he selectively treated his facial wound on his right flange with the plant juice (and no other body parts), (II) the behavior was repeated several times, not only plant juice but later also more solid plant material was applied until the wound was fully covered and (III) the entire process took a considerable amount of time. It is possible, that wound treatment with *Fibraurea tinctoria* emerges through accidental individual innovation^63^. Individuals may accidentally touch their wounds while feeding on *Fibraurea tinctoria* and thus unintentionally apply the plant’s juice to their wounds. As *Fibraurea tinctoria* has potent analgesic effects, individuals may feel an immediate pain release, causing them to repeat the behavior several times and subsequently apply solid plant matter possibly to also cover the wound as a protection against flies (as the case reported here suggests). Immature orangutans rely on observational social learning for the acquisition of their skill repertoires^64^ and recent evidence suggests that social learning continues into adulthood^65^. Therefore, given that it occurs frequent enough and in social contexts, wound treatment with *Fibraurea tinctoria* may also spread socially from individual to individual.

However, up to date, in 21 years and 28′000 observation hours, we never observed any other orangutans at Suaq using *Fibraurea tinctoria* to treat their wounds. On the one hand, this may be due to the fact that we rarely encounter injured orangutans at Suaq. Due to high food availability, high social tolerance between orangutans and relatively stable social hierarchies (each area is usually inhabited by a dominant male and several females^66^), there are few physical fights. However, during the time of this study, there was no clearly dominant male present in the research area. Rakus had just gone through his secondary sexual development in the year before the incident and, as a newly flanged male, he seemed to try to establish himself as the new dominant local male which is reflected in our behavioral data collected during this time. As a result of that, Rakus was involved in several long-call battles^66^ and physical altercations with other flanged males that were resident in and around the area at that time. On the other hand, it may be that wound treatment with *Fibraurea tinctoria* has so far been absent in the behavioral repertoire of the Suaq orangutan population. Like all adult males in the area, Rakus was not born in Suaq (his origin is unknown). Orangutan males disperse from their natal area during/after puberty over long distances to either establish a new home range in another area (mostly as dominant flanged male) or are moving between other’s home ranges (as unflanged males or flanged males)^67^. Therefore, any adult male in an area does not originate from the area^67^. Thus, as of now, it is impossible to find out where the males come from. Therefore, it is possible that the behavior is shown by more individuals in his natal population.

Of the few injured orangutans that we observed at Suaq so far, we observed one other instance of possible wound soothing/treatment behavior. Flanged male named Pluto repeatedly put his injured finger into the water of a pitcher plant. The water may have had a cooling effect that could eventually relieve pain or help clean the wound.

Taken together, chemical analyzes of the properties of the *Fibraurea tinctoria* and the orangutan's particular goal-oriented behavior are consistent with the hypothesis that the process of preparing and applying herbal ointments may be a form of self-medication that reduces pain, prevents inflammation, and accelerates wound healing. The present study may thus present the first report of active wound management with a biological active substance in a great ape species and provides new insights into the existence of self-medication in our closest relatives and in the evolutionary origins of wound medication more broadly. As forms of active wound treatment are not just a human universal but can also be found in both African and Asian great apes, it is possible that there exists a common underlying mechanism for the recognition and application of substances with medical or functional properties to wounds and that our last common ancestor already showed similar forms of ointment behavior.

### Supplementary Information

Supplementary Information 1.

Supplementary Video 1.

Supplementary Video 2.

## Data Availability

The data of this study consist of detailed pictures of the wounded orangutan and the specimen used by the orangutan to treat the wound. All data are provided in the manuscript.
