# Supplementary material for: Active self-treatment of a facial wound with a biologically active plant by a male Sumatran orangutan
Source: Sci Rep. 2024 May 2;14:8932. doi: 10.1038/s41598-024-58988-7 (PMC11066025; doi:10.1038/s41598-024-58988-7)
Supplement: Supplementary file 1 — Supplementary Information 1. [file 41598_2024_58988_MOESM1_ESM.docx]

**Supplementary information**

**Title:** Active wound treatment of a facial wound with a biologically active plant in a male Sumatran orangutan

**Authors:** Isabelle B. Laumer, Arif Rahman, Tri Rahmaeti, Ulil Azhari, Hermansyah, Sri Suci Utami Atmoko, Caroline Schuppli

1. **Resting time during wound healing**


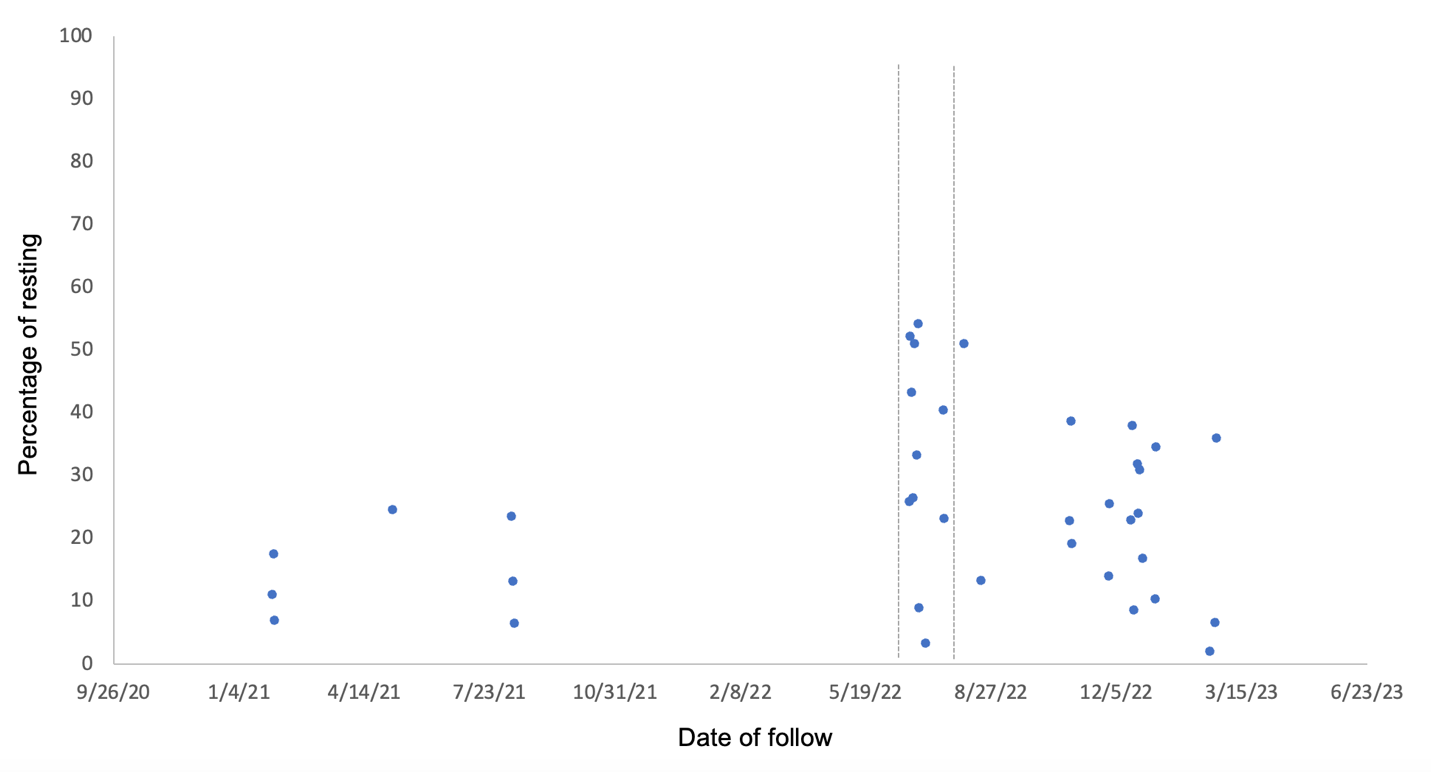


**Figure S1** Percentage of resting time between January 30, 2021, to February 22, 2023. The time from when he was found with the facial wound until the wound had fully closed (June 22, 2022 to July 20, 2023) is indicated by dashed lines. Data were generated from focal animal follows at two-minute intervals following standardized protocols.

**Table S1** Scientific name of the plant, synonyms, classification, source database and reference.

| **Accepted scientific name** | ***Fibraurea tinctoria* Lour.** |
| --- | --- |
| Synonyms | *Cocculus fibraurea* DC. |
|  | *Cocculus rimosus* Bl. |
|  | *Fibraurea chloroleuca* Miers |
|  | *Fibraurea fasciculata* Miers |
|  | *Fibraurea laxa* Miers |
|  | *Fibraurea manipurensis* Brace ex Diels |
|  | *Fibraurea trotteri* Watt |
|  | *Menispermum rimosum* Spreng. |
|  | *Menispermum tinctorium* Spreng. |
| Classification | *Plantae* |
| Phylum | *Tracheophyta* |
| Class | *Magnoliopsida* |
| Order | *Ranunculales* |
| Family | *Menispermaceae* |
| Genus | *Fibraurea* |
| Distribution | Java, Sumatra, peninsular Malaysia, Java, Borneo, Sulawesi, Philippines (Dinagat), Manipur, Thailand, Indochina, Myanmar [Burma] (Taninthayi), S-China, Andamans (Little Andaman Isl.), Nicobars (Central Nicobars, Great Nicobar Isl., Little Nicobar Isl.), Vietnam, Laos |
| Source database | *World Plants, March 2014* |
|  | <http://www.catalogueoflife.org/annual-checklist/2014/details/species/id/16603984/source/tree> |
| Reference | [Roskov Y.; Kunze T.; Orrell T.; Abucay L.; Paglinawan L.; Culham A.; Bailly N.; Kirk P.; Bourgoin T.; Baillargeon G.; Decock W.; De Wever A.; Didžiulis V., eds. (2014). "Species 2000 & ITIS Catalogue of Life: 2014 Annual Checklist". Species 2000: Reading, UK. Retrieved 12 January 2018.](http://www.catalogueoflife.org/annual-checklist/2014/browse/tree/id/17277402) |

**Movie S1** Videos of male orangutan Rakus on the day as the wound was first observed, on the day he treated his wound with *Fibraurea tinctoria*, on the day after as he was feeding on *Fibraurea tinctoria*, and four and five days after wound treatment.

**Movie S2** Male orangutan Rakus emitting a long call on the day the wound was first observed. His wound on his right flange and inside his mouth are visible.
